# Supplementary material for: Water as a gas separation membrane
Source: Nat Commun. 2026 Mar 23;17:4311. doi: 10.1038/s41467-026-70630-w (PMC13172504; doi:10.1038/s41467-026-70630-w)
Supplement: Supplementary file 1 — Supplementary Information [file 41467_2026_70630_MOESM1_ESM.pdf]

Supplementary Information

Water as a gas separation membrane

Nature Communications

Kian P. Lopez[1], Max Saffer-Meng[1], Mohammad Allouzi[2,3], Yukai Tomsovic[2,4], Joshua N. Sherrit[1], Sasha R. Neefe[1], Patrick O. Saboe[5], Mou Paul[5], Abhishek Roy[5], and Anthony P. Straub[2,3,4*]

[1]Department of Chemical & Biological Engineering, University of Colorado Boulder, Boulder, CO 80303, USA
[2]Department of Mechanical and Process Engineering, ETH Zürich, Zürich 8092, Switzerland
[3]Department of Civil, Environmental & Architectural Engineering, University of Colorado Boulder, Boulder, CO 80303, USA
[4]Materials Science & Engineering Program, University of Colorado Boulder, Boulder, CO 80303, USA
[5]National Laboratory of the Rockies, Golden, CO, 80401, USA

[*]Corresponding author:
Anthony Straub, Email: astraub@ethz.ch

Supplementary Note 1 | Design of membranes with thin water layers

In addition to testing commercially available alumina and polymer substrates, we fabricated high performance liquid water membranes with thin water layers. The design criteria for high performance liquid water membranes are as follows:

1. To resist water displacement by gas under high applied pressure, pores must be hydrophilic and have sub-100-nm pore diameters. These small pores generate high capillary pressures that stabilize the water layer against external pressure, in accordance with Young-Laplace theory (equation (4)), which states that liquid displacement pressure scales with the inverse of pore diameter[1,2].
2. To enable high gas permeances, the liquid water layer must also be thin (less than 1 μm) to minimize diffusion resistances as dissolved gases transport from the feed gas-liquid interface to the permeate side (equation (2)).
3. The membrane must be made from materials mechanically robust under high applied gas pressures.

Following these criteria, high-performance membranes were fabricated using isoporous anodic aluminum oxide (AAO) substrates modified to trap water in a thin hydrophilic region of tunable length (see Methods). The natively hydrophilic, 40-nm-diameter alumina nanopores were first modified using a hydrophobic coating via chemical vapor deposition of a fluorosilane molecule onto the native hydroxyl groups on the alumina surface (Supplementary Fig. 1 and 2). Sub-micron lengths of the upper pore were then coated using metal sputtering where the sputtering angle controlled the penetration depth. The depth of the sputtered platinum layer, which is approximately equivalent to the water layer thickness, l, was estimated using equation (S1):

$$l = \frac{a}{\tan(90-\theta)} \qquad \text{(S1)}$$

where a is pore diameter, and θ is the incident angle from the membrane surface. As described in our previous work[3], decreasing the incident angle, θ, or the membrane's pore diameter, a, allows for a decrease in the penetration depth of the sputtered platinum. The metal surface was then selectively modified using thioglycolic acid (Supplementary Fig. 3) to render the surface hydrophilic, enabling the retention of a stable water-trapping layer.

Supplementary Note 2 | Characterization of fabricated membranes

Electron imaging was used to verify that the fabricated membranes achieved the desired structure. Scanning transmission electron micrographs of membrane cross sections showed that the pores had a uniform, cylindrical structure (Supplementary Fig. 4, 5, and 6). Elemental mapping using energy dispersive spectroscopy further demonstrated that the platinum metal layer applied by sputtering is concentrated in the upper sub-200 nm of the membrane, with minimal penetration into the pore, which clearly showed the presence of aluminum (Supplementary Fig. 5 and 6). The thickness of the sputtered metal layer in electron imaging showed agreement with geometric calculations for the hydrophilic layer thickness based on the sputtering angle (Supplementary Table 1).

Chemical modification of the membrane surface was confirmed using contact angle measurements and surface chemical analysis. The fabricated membranes exhibited a highly hydrophilic top surface, where water droplets rapidly wicked into the membrane, as expected based on the metal sputtering and hydrophilic thioglycolic acid modification, which had a measured intrinsic contact angle of 5.3° (Supplementary Fig. 7). In contrast, the bottom surface of the membrane that was modified using fluorosilanes was superhydrophobic with a water contact angle of 162° (Supplementary Fig. 8). X-ray photoelectron spectroscopy (XPS) analysis verified the presence of expected chemical elements from the surface modifications (Supplementary Fig. 9). The hydrophobic bottom layer showed a strong F 1s peak at 688.5 eV, characteristic of C–F bonds, confirming successful fluorosilane functionalization. Platinum sputtered onto the top surface was confirmed by the presence of Pt 4f and Pt 4d peaks at 74.6 eV and 315.0 eV, respectively. The hydrophilic top surface was functionalized with thioglycolic acid, as confirmed by the presence of sulfur peaks at 164.0 eV (S 2p) and 228.0 eV (S 2s). The C 1s envelope further supported the presence of $C–F_2$ and $C–F_3$ bonds on the hydrophobic surface and carboxylic acid groups (–COOH) on the hydrophilic surface (Supplementary Fig. 10).

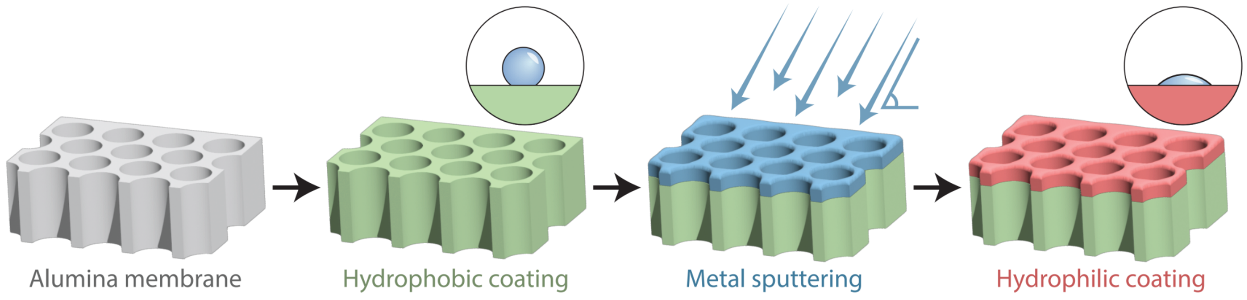

Supplementary Fig. 1 | Schematic of the membrane fabrication procedure where membranes are (i) modified using a hydrophobic coating, (ii) sputtered with metal at a controlled angle, and (iii) modified using a hydrophilic thiol that selectively coats the metal layer.

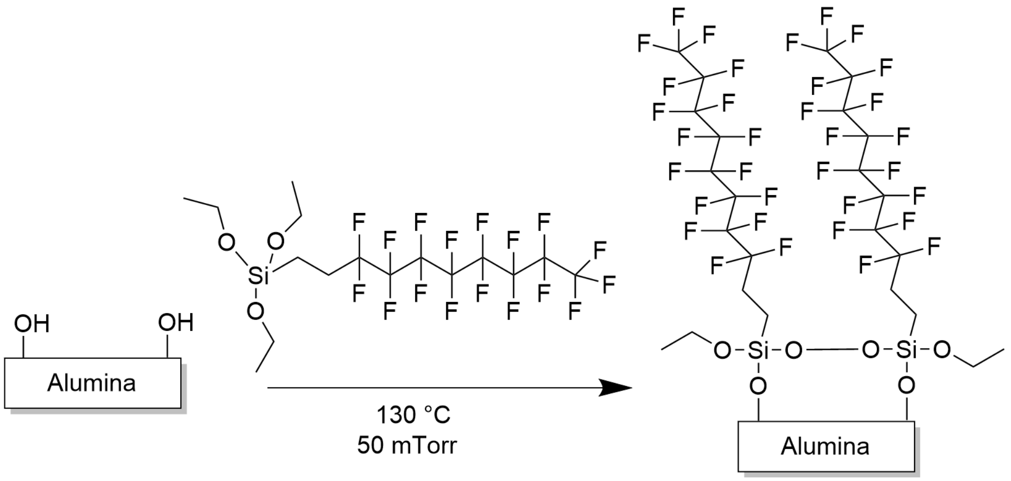

Supplementary Fig. 2 | Reaction 1H,1H,2H,2H-perfluorodecyltriethoxysilane on the anodic aluminum oxide (AAO) membrane surface to create a hydrophobic surface coating.

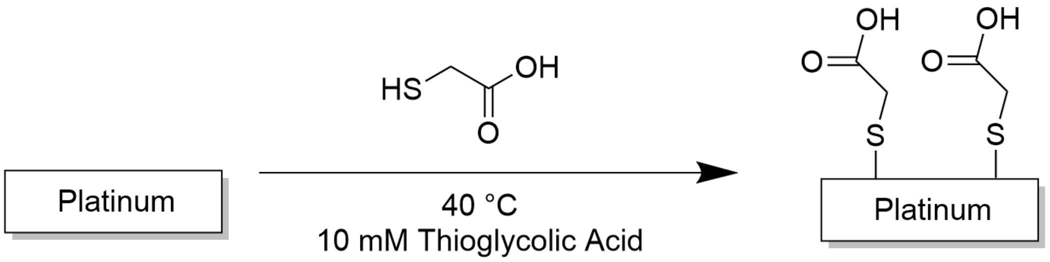

Supplementary Fig. 3 | Reaction of thioglycolic acid on the sputtered platinum surface to create a hydrophilic surface layer.

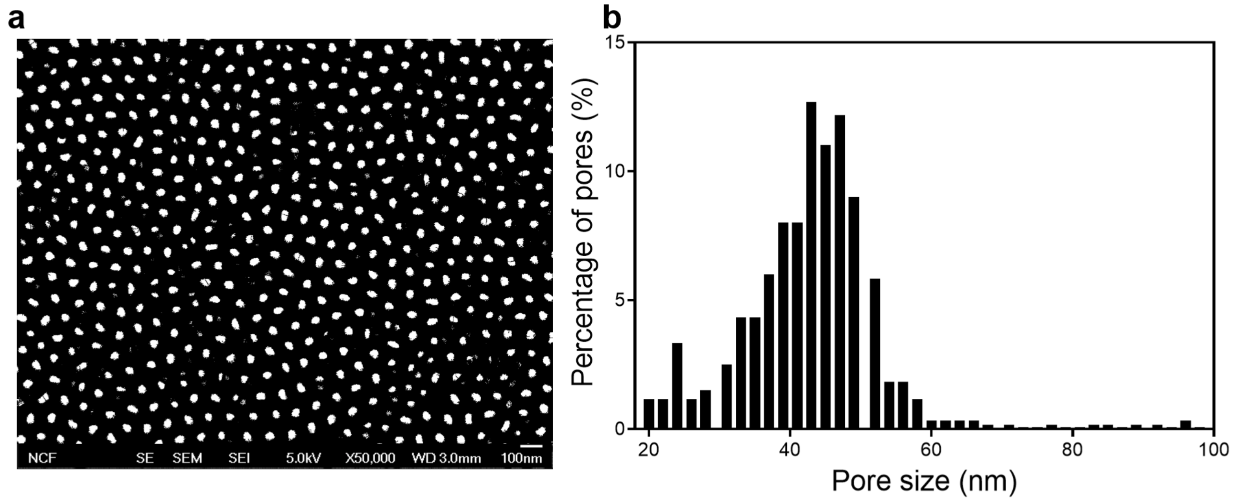

Supplementary Fig. 4 | Binary SEM image of 40 nm pore size AAO membrane (**a**) and pore size distribution analyzed with ImageJ (**b**).

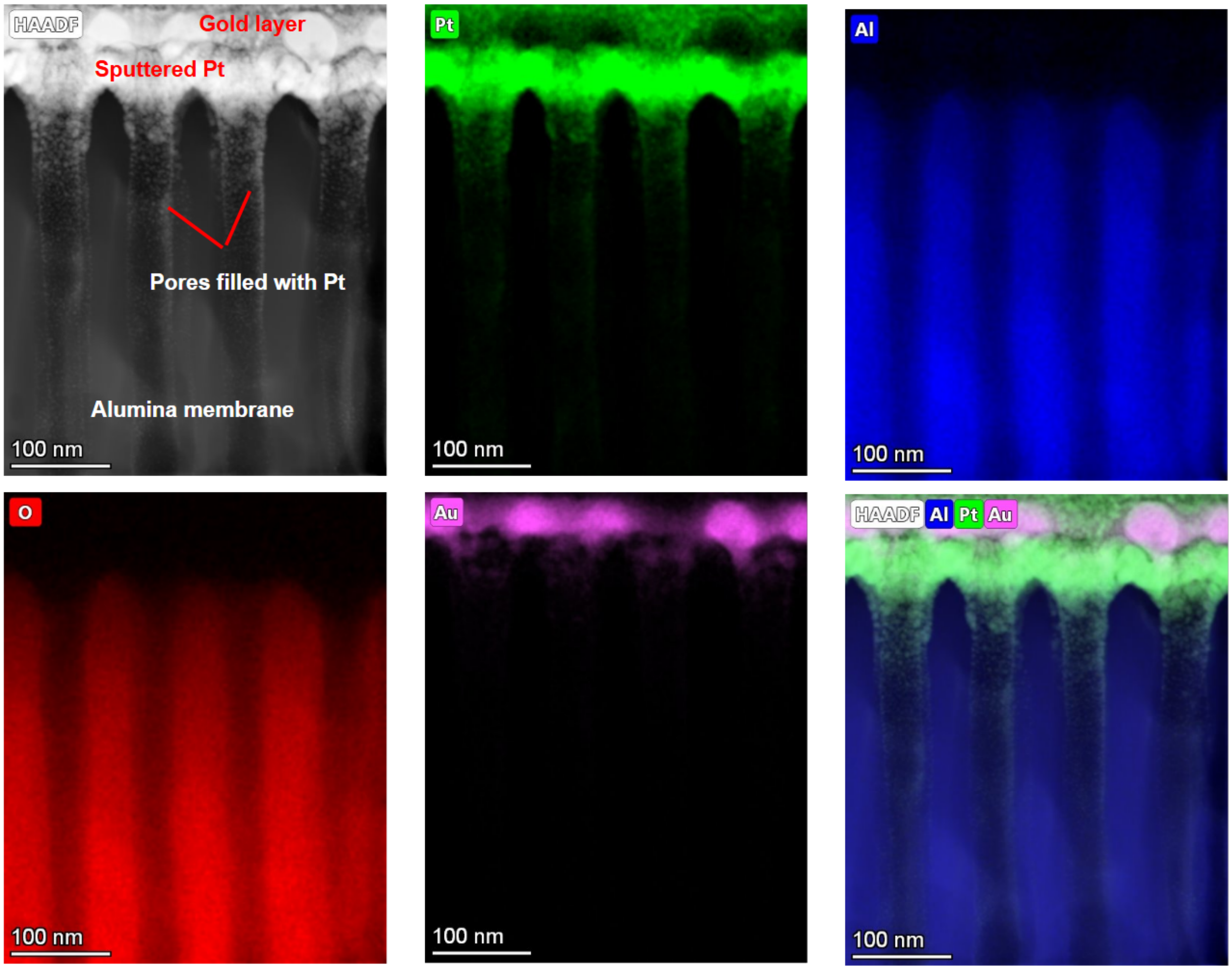

Supplementary Fig. 5 | Scanning transmission electron microscopy (STEM) high-angle annular dark-field image and element mapping images using energy dispersive spectroscopy (EDS). Images show the cross section of a 40 nm pore diameter AAO membrane sample sputtered with platinum. EDS elemental mapping is used to show the presence of platinum on the top surface of the AAO membrane as well as oxygen and aluminum within the membrane itself.

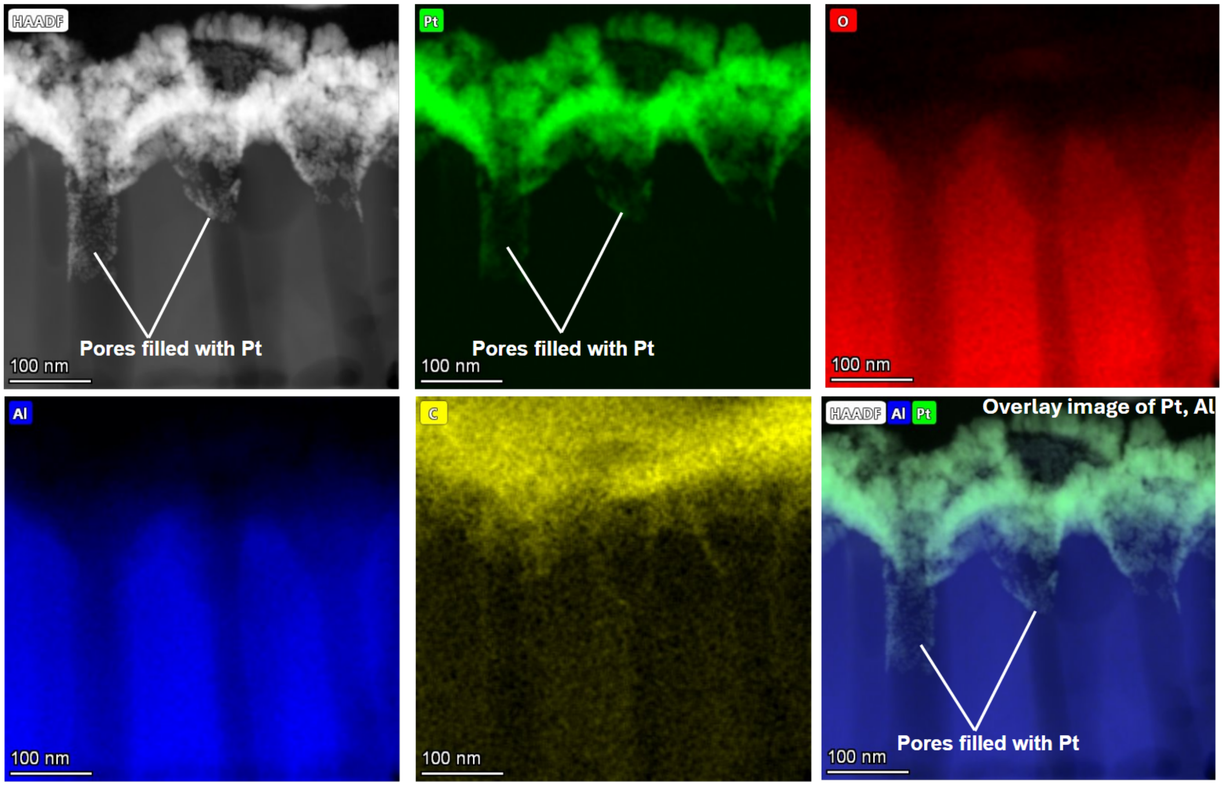

Supplementary Fig. 6 | STEM with EDS images for 80 nm pore diameter AAO membrane samples sputtered with platinum. Images show the cross section of an 80 nm pore diameter AAO membrane sample sputtered with platinum. EDS elemental mapping is used to show the presence of platinum on the top surface of the AAO membrane as well as oxygen and aluminum within the membrane itself.

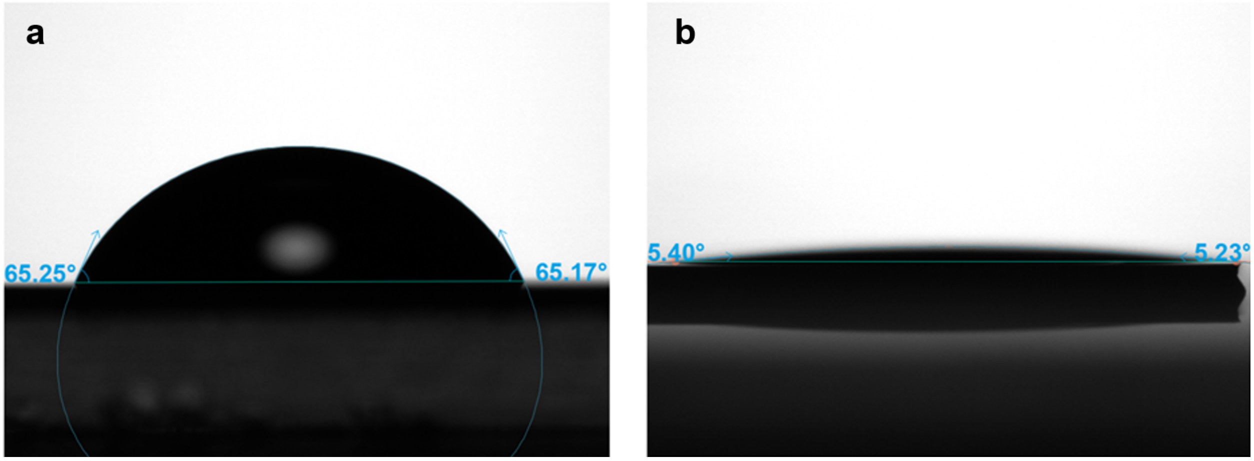

Supplementary Fig. 7 | Static water contact angle measurements of (**a**) unmodified silicon wafer and (**b**) a silicon wafer sputtered with platinum and modified with hydrophilic thioglycolic acid.

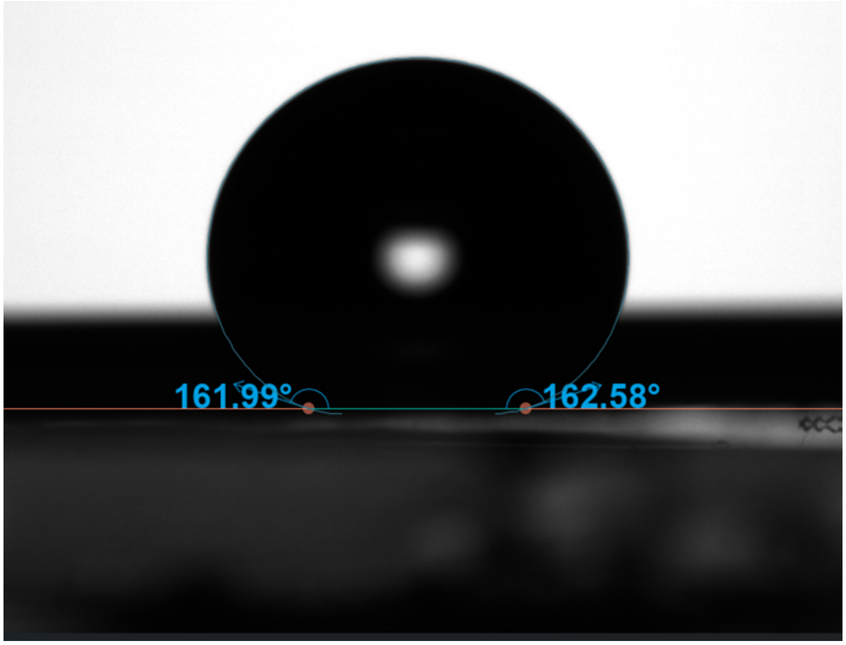

Supplementary Fig. 8 | Static water contact angle measurement of anodic aluminum oxide modified with 1H,1H,2H,2H-perfluorodecyltriethoxysilane.

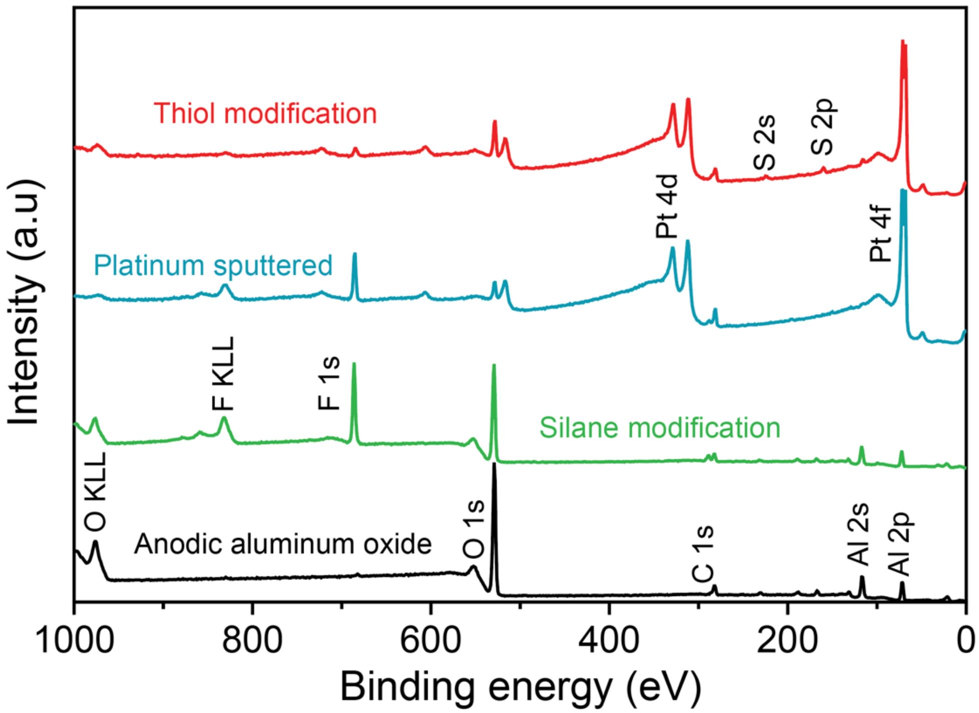

Supplementary Fig. 9 | X-ray photoelectron spectra taken for the pristine alumina membrane, the fluorosilane modified membrane, the platinum-sputtered surface, and the thiol-modified platinum.

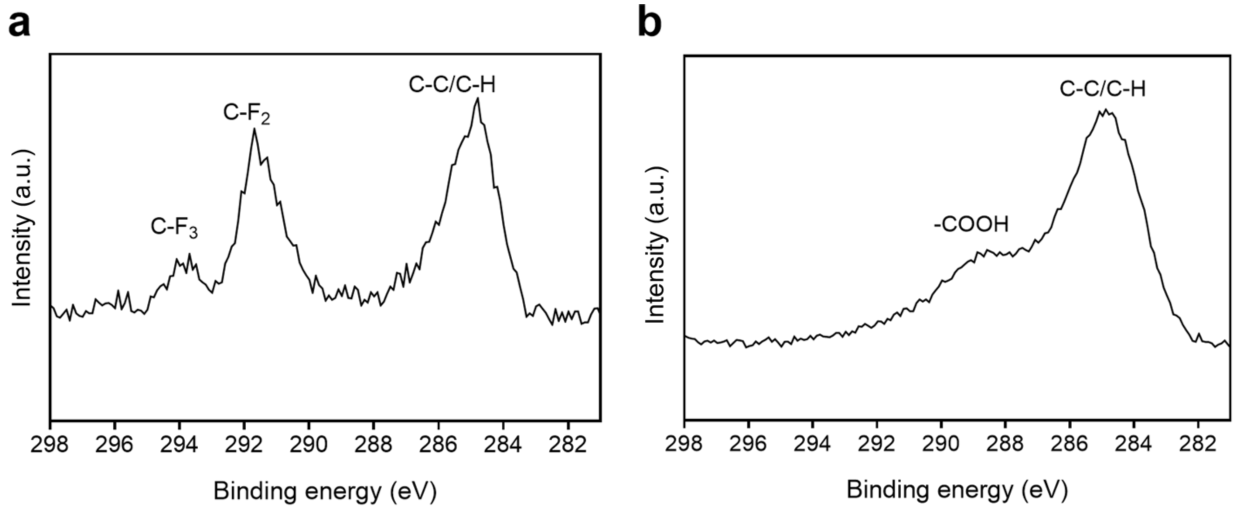

Supplementary Fig. 10 | High resolution X-ray photoelectron spectra of C 1s peaks after (**a**) hydrophobic fluorosilane modification and (**b**) hydrophilic thiol modification.

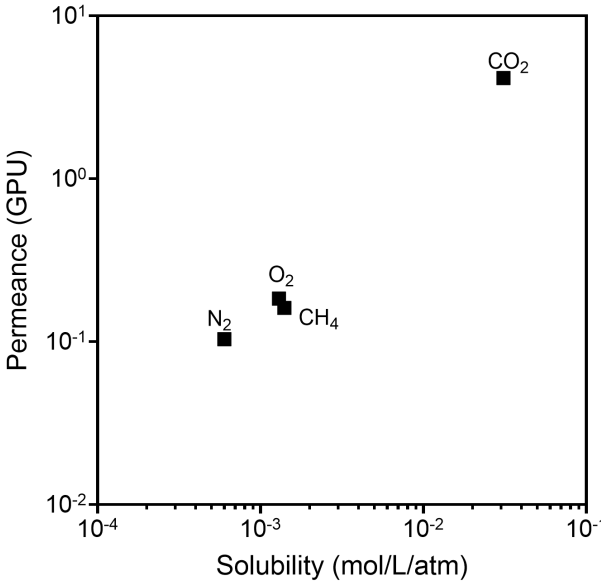

Supplementary Fig. 11 | N_2, O_2, CH_4, and CO_2 permeance across an unmodified 50 µm thick AAO membrane. Membranes were tested at 6.9 bar and 22 ± 2 °C.

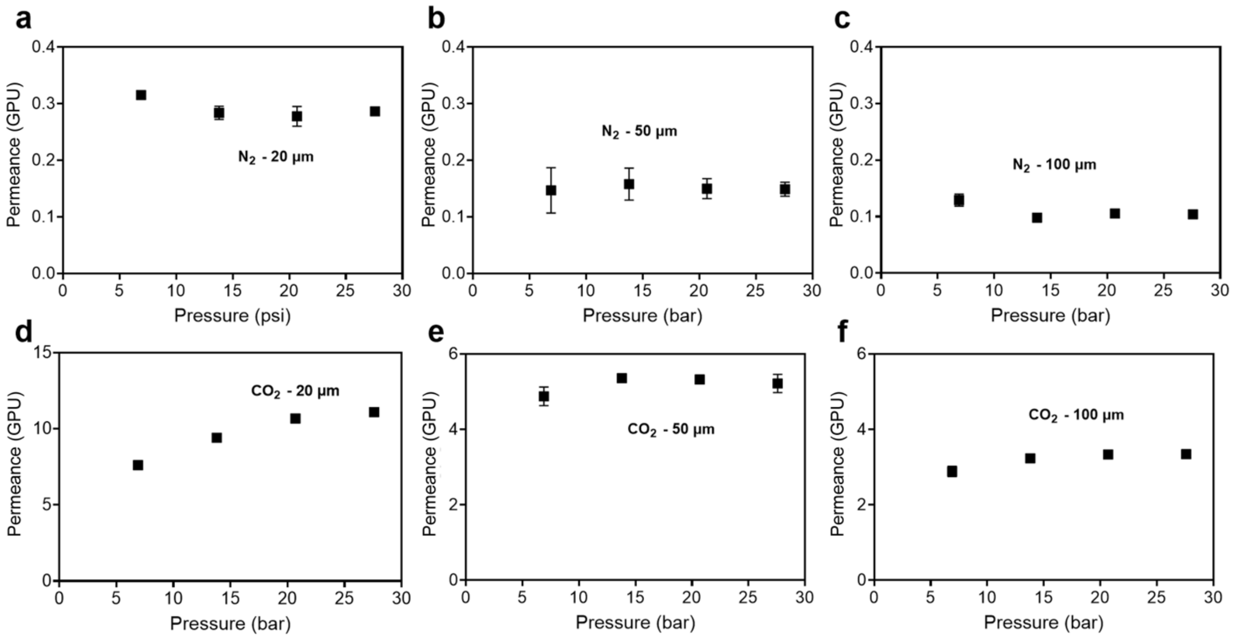

Supplementary Fig. 12 | N_2 permeance as a function of feed gas pressure for unmodified (**a**) 20, (**b**) 50, and (**c**) 100 µm thick AAO membranes. CO_2 permeance for unmodified (**d**) 20, (**e**) 50, and (**f**) 100 µm thick AAO membranes all with pore diameters of 40 nm. All gas permeation experiments were conducted at a temperature of 22 ± 2 °C.

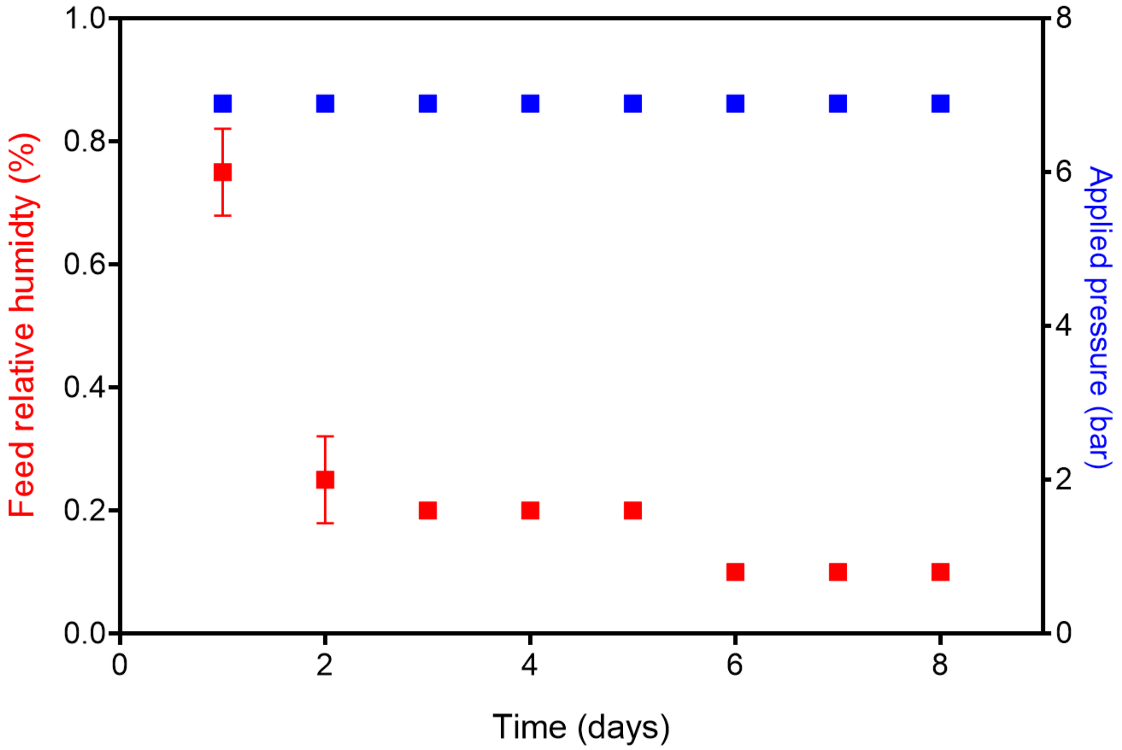

Supplementary Fig. 13 | Feed relative humidity and applied pressure over 8 days of operation. Relative humidity in the feed was limited to less than 1% by continuously venting feed gas. Temperature was maintained at 22 ± 2 °C. The gas flowrate was determined by measuring the displacement of a column of water on the permeate side of the membrane. In all plots, error bars denote the mean ±1 s.d. from at least three measurements.

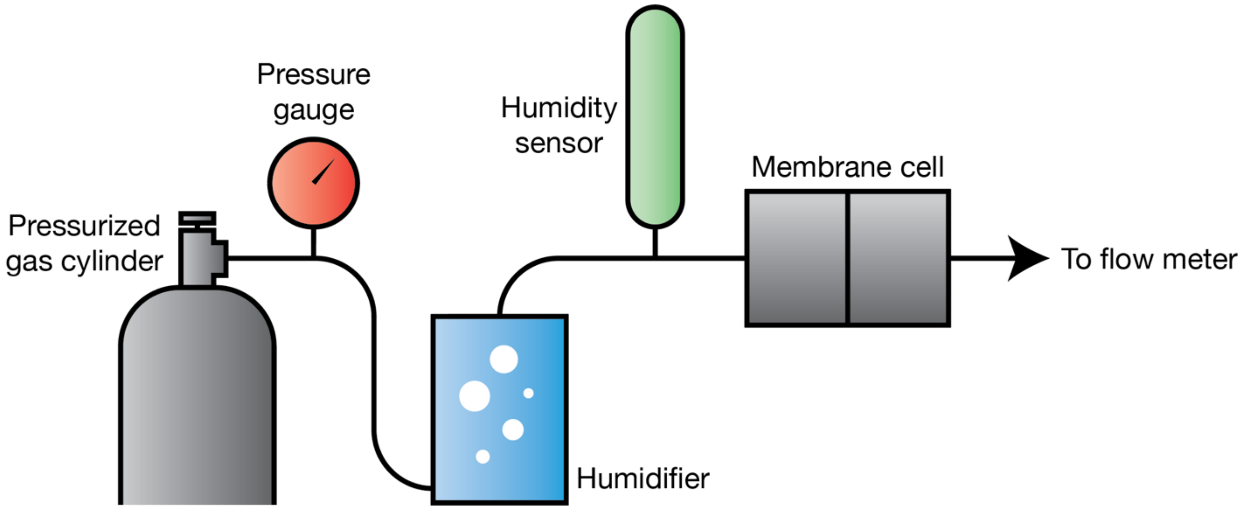

Supplementary Fig. 14 | Experimental setup for gas permeance and long-term stability testing.

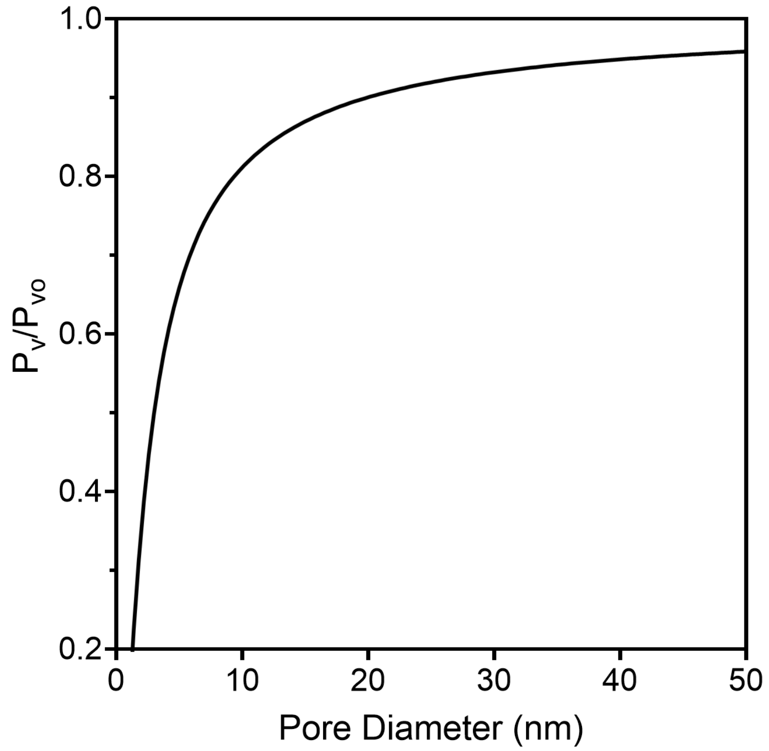

Supplementary Fig. 15 | Reduction in equilibrium vapor pressure from the Kelvin effect for pore diameters less than 50 nm.

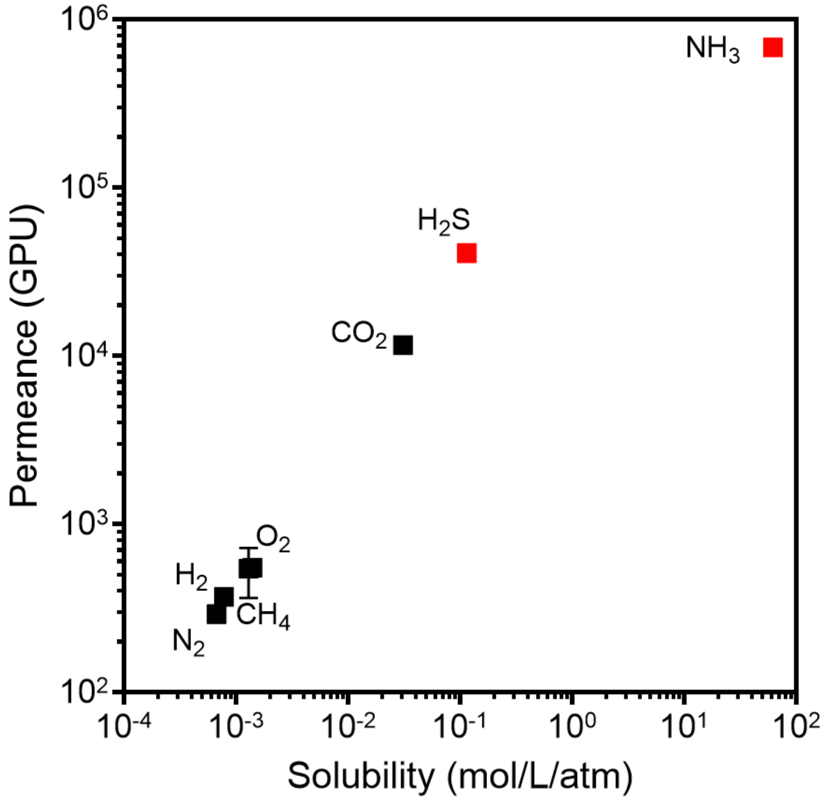

Supplementary Fig. 16 | Modeled permeance values for H_2S and NH_3 relative to experimentally measured gases.

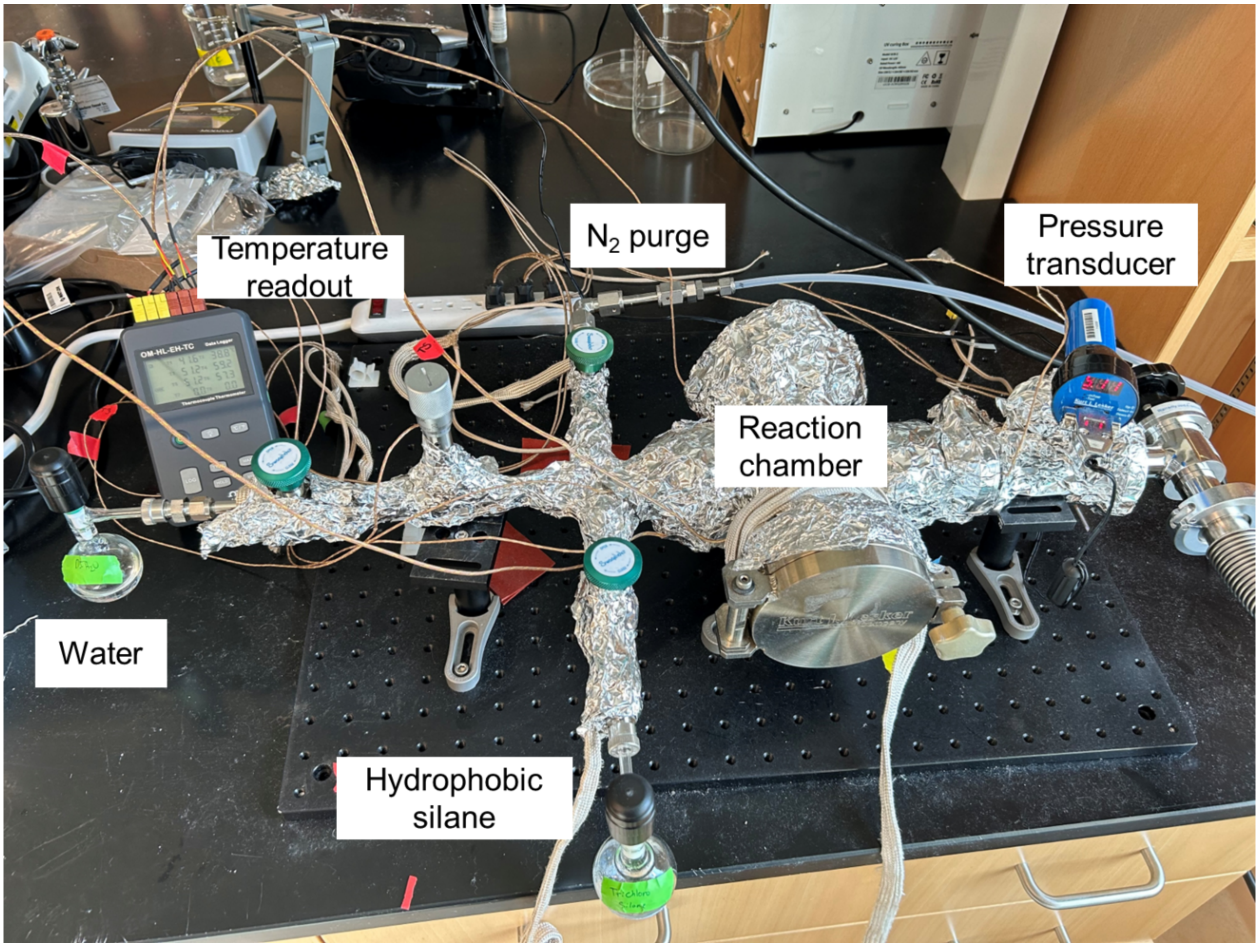

Supplementary Fig. 17 | Chemical vapor deposition (CVD) chamber used for hydrophobic silane modifications.

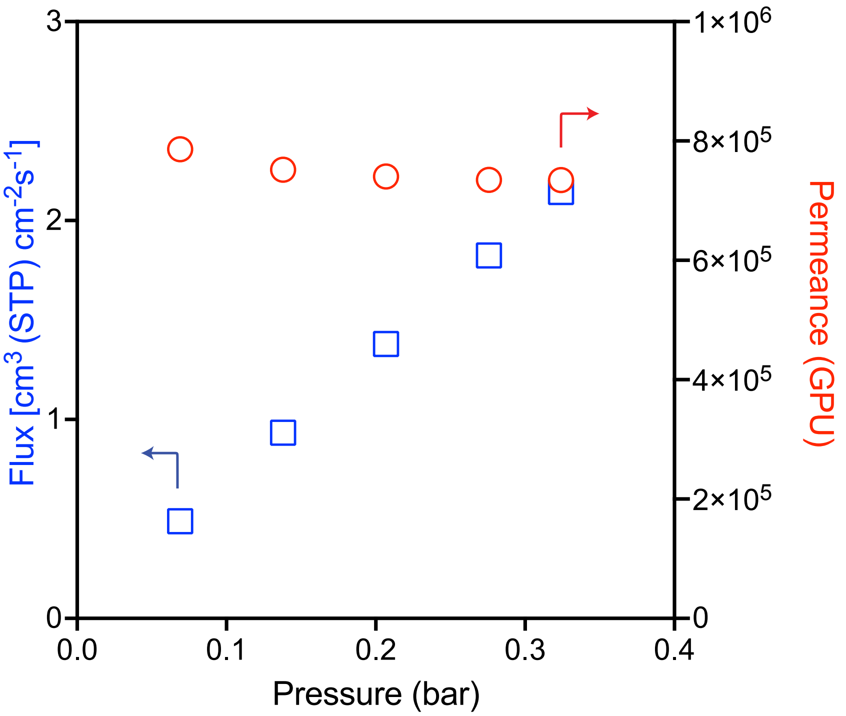

Supplementary Fig. 18 | N_2 flux and permeance measured across 50 µm thick AAO with 40 nm diameter pores with no water layer. Permeance is normalized to 12% porosity.

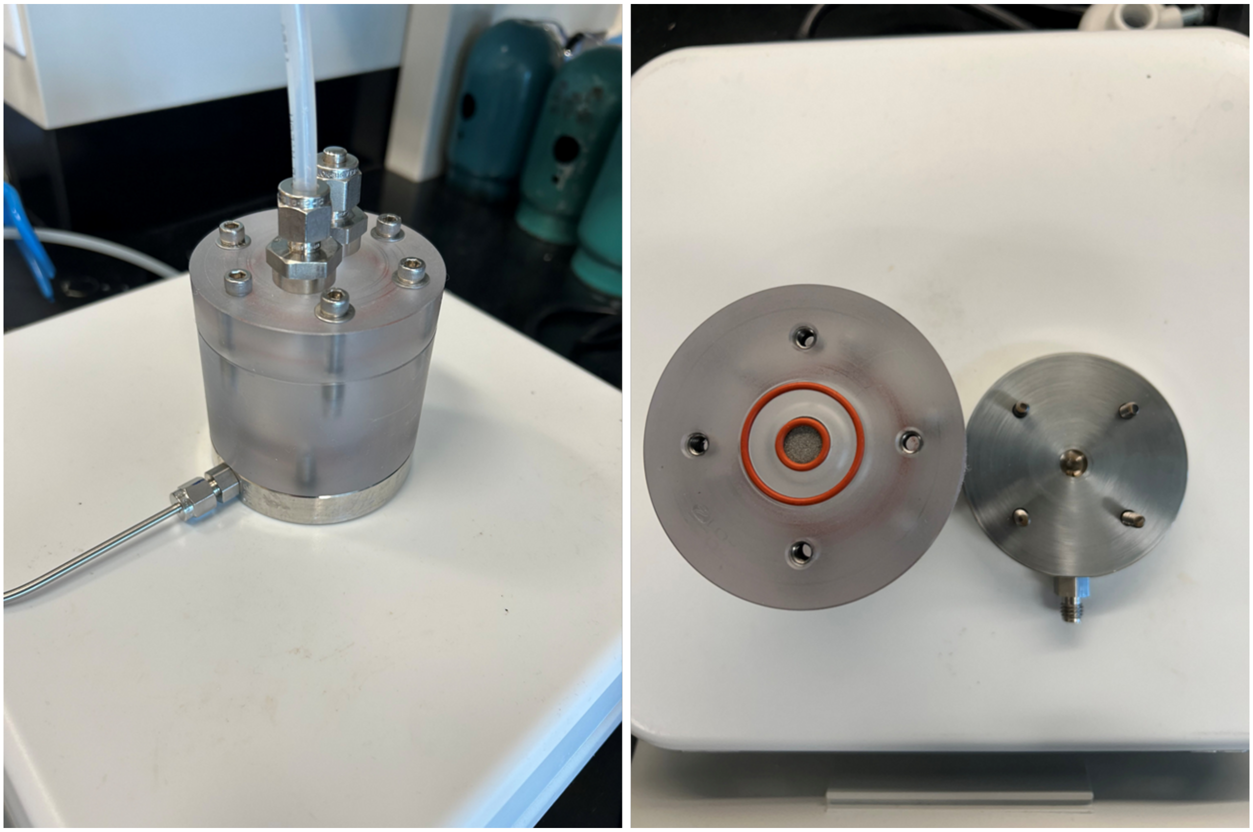

Supplementary Fig. 19 | Custom-made membrane cell for high pressure (75 bar) gas permeation testing. Membrane cell mounted a 13 mm diameter membrane sample. Pressurized feed gas entered through the bottom of the cell.

Supplementary Tables

Supplementary Table 1. Hydrophilic layer thickness estimation.

Pore diameter (nm)	Incident angle	Calculated thickness (nm)	Estimated thickness from STEM-EDS chemical mapping (nm)
80	65°	172	180 ± 12
40	80°	227	230 ± 17

Supplementary Table 2. Gas permeance values as measured and normalized to the 12% membrane porosity.

	CO_2 Permeance (GPU)	N_2 Permeance (GPU)	O_2 Permeance (GPU)	CH_4 Permeance (GPU)	H_2 Permeance (GPU)
Measured	1,390	34.9	65.1	66.1	44.3
Normalized to active area	11,600	291	543	551	369

Supplementary Table 3. Gas solubility and diffusion in water.

Species	Formula	K_h (M/atm)	Diffusion coefficient (cm^2/s)	Temperature
Carbon dioxide	CO_2	0.0334 (Ref[4])	1.91 x 10^{-5} (Ref[5])	25°C
Nitrogen	N_2	0.00067 (Ref[4])	2.00 x 10^{-5} (Ref[6])	25°C
Oxygen	O_2	0.00138 (Ref[4])	2.10 x 10^{-5} (Ref[7])	25°C
Methane	CH_4	0.0015 (Ref[4])	1.88 x 10^{-5} (Ref[8])	25°C
Hydrogen	H_2	0.00078 (Ref[4])	4.3 x 10^{-5} (Ref[9])	25°C
Hydrogen sulfide	H_2S	0.115 (Ref[4])	1.93 x 10^{-5} (Ref[10])	25°C
Ammonia	NH_3	62 (Ref[4])	1.54 x 10^{-5} (Ref[11])	20°C

Supplementary Table 4. Vapor pressure reduction from decreasing radius of curvature in nanoscale pores.

Pore Diameter (nm)	Minimum Radius of Curvature (nm)	Deviation from Equilibrium Vapor Pressure
2	1.02	0.64
5	2.54	0.34
10	5.08	0.19
20	10.15	0.10
50	25.39	0.04
100	50.77	0.02

Supplementary Table 5. Comparison of CO_2 permeance and selectivity from other membrane studies in the literature.

CO_2 Permeance (GPU)	CO_2:N_2 Selectivity	CO_2:CH_4 Selectivity	CO_2:H_2 Selectivity	Reference
1460	60	-	-	T. Brinkmann., et al.[12]
1000	50	-	-	L. S. White., et al.[13]
1650	50	-	-	L. S. White., et al.[13]
6820	17.9	-	-	Bushell A.F., et al.[14]
8268	25.1	-	-	B. Zhu., et al.[15]
1727	24.0	-	-	Ghalei B., et al.[16]
2952	26.9	-	-	Ghalei B., et al.[16]
5540	25.2	10.9	-	G. He., et al.[17]
6290	20.4	9.3	-	G. He., et al.[17]
4420	24.8	10.0	-	G. He., et al.[17]
3730	28.9	-	-	G. He., et al.[17]
9420	-	12.3	-	Wang Z., et al.[18]
8283	-	12.3	-	J. Lu., et al.[19]

13564	-	11.9	-	B. Zhu., et al.[15]
8268	-	18.7	-	B. Zhu., et al.[15]
410	-	-	10.2	S.R. Reijerkerk., et al.[20]
546	-	-	10.4	S.R. Reijerkerk., et al.[20]
682	-	-	10.5	S.R. Reijerkerk., et al.[20]
846	-	-	10.7	S.R. Reijerkerk., et al.[20]
1469	-	-	9.7	C.H. Lau., et al.[21]
1589	-	-	10.2	C.H. Lau., et al.[21]
1863	-	-	10.5	C.H. Lau., et al.[21]
531	-	-	12.1	H. Lin., et al[22]
491	-	-	11.3	H. Lin., et al[22]
300	-	-	10.2	H. Lin., et al[22]
237	-	-	9.4	H. Lin., et al[22]
142	-	-	7.9	H. Lin., et al[22]
1874	-	-	12	Polaris[23]
513	170			CMS[24]
11616	39.9	26	31.4	This work

Supplementary Table 6. Commercial membrane properties assuming a porosity of 60% and a tortuosity of 2.

Membrane	Measured Membrane Thickness (µm)	Calculated Water Layer Thickness (µm)	Displacement Pressure (bar)	Specified Pore Size from Supplier (nm)	Calculated Maximum Pore Diameter (nm)
PVDF	110	116	1.5	450	960
PES	140	97	6.6	30	218

References

1. Zha, F. F., Fane, A. G., Fell, C. J. D. & Schofield, R. W. Critical displacement pressure of a supported liquid membrane. *Journal of Membrane Science* **75**, 69–80 (1992).
2. Liu, H. & Cao, G. Effectiveness of the Young-Laplace equation at nanoscale. *Sci Rep* **6**, 23936 (2016).
3. Nguyen, D. T., Lee, S., Lopez, K. P., Lee, J. & Straub, A. P. Pressure-driven distillation using air-trapping membranes for fast and selective water purification. *Science Advances* **9**, eadg6638 (2023).
4. Sander, R. Compilation of Henry's law constants (version 5.0.0) for water as solvent. *Atmospheric Chemistry and Physics* **23**, 10901–12440 (2023).
5. Polat, H. M. *et al.* Diffusivity of CO2 in H2O: A Review of Experimental Studies and Molecular Simulations in the Bulk and in Confinement. *J. Chem. Eng. Data* **69**, 3296–3329 (2024).
6. Cadogan, S. P., Maitland, G. C. & Trusler, J. P. M. Diffusion Coefficients of CO2 and N2 in Water at Temperatures between 298.15 K and 423.15 K at Pressures up to 45 MPa. *J. Chem. Eng. Data* **59**, 519–525 (2014).
7. Xing, W. *et al.* 1 - Oxygen Solubility, Diffusion Coefficient, and Solution Viscosity. in *Rotating Electrode Methods and Oxygen Reduction Electrocatalysts* (eds Xing, W., Yin, G. & Zhang, J.) 1–31 (Elsevier, Amsterdam, 2014). doi:10.1016/B978-0-444-63278-4.00001-X.
8. Witherspoon, P. A. & Saraf, D. N. Diffusion of Methane, Ethane, Propane, and n-Butane in Water from 25 to 43°. *J. Phys. Chem.* **69**, 3752–3755 (1965).
9. Wang, S., Zhou, T., Pan, Z. & Trusler, J. P. M. Diffusion Coefficients of N2O and H2 in Water at Temperatures between 298.15 and 423.15 K with Pressures up to 30 MPa. *J. Chem. Eng. Data* **68**, 1313–1319 (2023).
10. Tamimi, A., Rinker, E. B. & Sandall, O. C. Diffusion Coefficients for Hydrogen Sulfide, Carbon Dioxide, and Nitrous Oxide in Water over the Temperature Range 293-368 K. *J. Chem. Eng. Data* **39**, 330–332 (1994).
11. Rives, R., Salavera, D., Campos, J. & Coronas, A. Development of optical digital interferometry for visualizing and modelling the mass diffusion of ammonia in water in an absorption process. *Experimental Thermal and Fluid Science* **130**, 110509 (2022).
12. Brinkmann, T. *et al.* Development of CO2 Selective Poly(Ethylene Oxide)-Based Membranes: From Laboratory to Pilot Plant Scale. *Engineering* **3**, 485–493 (2017).
13. White, L. S., Amo, K. D., Wu, T. & Merkel, T. C. Extended field trials of Polaris sweep modules for carbon capture. *Journal of Membrane Science* **542**, 217–225 (2017).
14. Bushell, A. F. *et al.* Gas permeation parameters of mixed matrix membranes based on the polymer of intrinsic microporosity PIM-1 and the zeolitic imidazolate framework ZIF-8. *Journal of Membrane Science* **427**, 48–62 (2013).

15. Zhu, B. *et al.* Boosting membrane carbon capture via multifaceted polyphenol-mediated soldering. *Nat Commun* **14**, 1697 (2023).

16. Ghalei, B. *et al.* Enhanced selectivity in mixed matrix membranes for CO2 capture through efficient dispersion of amine-functionalized MOF nanoparticles. *Nat Energy* **2**, 1–9 (2017).

17. He, G. *et al.* High-permeance polymer-functionalized single-layer graphene membranes that surpass the postcombustion carbon capture target. *Energy & Environmental Science* **12**, 3305–3312 (2019).

18. Wang, Z., Ren, H., Zhang, S., Zhang, F. & Jin, J. Polymers of intrinsic microporosity/metal–organic framework hybrid membranes with improved interfacial interaction for high-performance CO2 separation. *J. Mater. Chem. A* **5**, 10968–10977 (2017).

19. Lu, J. *et al.* Preparation of Amino-Functional UiO-66/PIMs Mixed Matrix Membranes with [bmim][Tf2N] as Regulator for Enhanced Gas Separation. *Membranes (Basel)* **11**, 35 (2021).

20. Reijerkerk, S. R., Knoef, M. H., Nijmeijer, K. & Wessling, M. Poly(ethylene glycol) and poly(dimethyl siloxane): Combining their advantages into efficient CO2 gas separation membranes. *Journal of Membrane Science* **352**, 126–135 (2010).

21. Lau, C. H. *et al.* Silica Nanohybrid Membranes with High CO2 Affinity for Green Hydrogen Purification. *Advanced Energy Materials* **1**, 634–642 (2011).

22. Lin, H. *et al.* Transport and structural characteristics of crosslinked poly(ethylene oxide) rubbers. *Journal of Membrane Science* **276**, 145–161 (2006).

23. Kniep, J. *et al.* *FIELD TESTS OF MTR MEMBRANES FOR SYNGAS SEPARATIONS: Final Report of CO2-Selective Membrane Field Test Activities at the National Carbon Capture Center*. (2017).

24. Rahimalimamaghani, A., Ramezani, R., Tanaka, D. A. P. & Gallucci, F. Carbon Molecular Sieve Membranes for Selective CO2/CH4 and CO2/N2 Separation: Experimental Study, Optimal Process Design, and Economic Analysis. *Ind. Eng. Chem. Res.* **62**, 19116–19132 (2023).
